# The Evaluation of Remote Monitoring Technology Across Participants With Different Skin Tones

**DOI:** 10.7759/cureus.45075

**Published:** 2023-09-12

**Authors:** Debjyoti Talukdar, Luis Felipe De Deus, Nikhil Sehgal

**Affiliations:** 1 Department of Medical Research, Mkhitar Gosh Armenian-Russian International University, Yerevan, ARM; 2 Department of AI Research, Vastmindz Limited, London, GBR

**Keywords:** remote photoplethysmography, heart rate, cardiac activity, blood pressure, respiration rate, bioelectrical impedance analysis, heart rate variability (hrv), vital sign monitoring, artificial intelligence, skin tone

## Abstract

Background: Many research studies seek to improve vital sign monitoring to enhance the conditions under which doctors and caregivers track patients' health. Non-invasive and contactless monitoring has emerged as an optimal solution for this problem, with telemedicine, self-monitoring, and well-being tools being the next generation of technology in the biomedical field. However, there is worldwide concern about the general purpose and bias toward a certain demographic group of these techniques. In particular, skin tone and the accuracy of monitoring dark skin tone groups have been key questions among researchers, with the lack of results and studies contributing to this uncertainty.

Methods: This paper proposes a benchmark for remote monitoring solutions against a medical device across different skin tone people. Around 330 videos from 90 patients were analyzed, and heart rate (HR) and heart rate variability (HRV) were compared across different subgroups. The Fitzpatrick scale (1-6) was used to classify participants into three skin tone groups: 1 and 2, 3 and 4, and 5 and 6.

Results: The results showed that our proposed methodology could estimate heart rate with a mean absolute error of 3 bpm across all samples and subgroups. Moreover, for heart rate variability (HRV) metrics, we achieved the following results: in terms of mobility assistive equipment (MAE), the HRV-inter-beat interval (IBI) was 10 ms, the HRV-standard deviation of normal to normal heartbeats (SDNN) was 14 ms, and the HRV-root mean square of successive differences (RMSSD) between normal heartbeats was 22 ms. No significant performance decrease was found for any skin tone group, and there was no error trend toward a certain group.

Conclusions: The study showed that our methodology meets acceptable agreement levels for the proposed metrics. Furthermore, the experiments showed that skin tone did not impact the results, which remained within the same range across all groups. Moreover, it enables the end users to understand their general well-being and improve their overall health.

## Introduction

Non-invasive vital sign monitoring is an important aspect of healthcare as it allows doctors and caregivers to track a patient's vital signs without the need for invasive procedures. Established methods with the help of contact sensors such as photoplethysmography (PPG), bioelectrical impedance analysis (BIA), and electrocardiogram (ECG) can be used to capture physiological data. These methods can measure several different physiological parameters, such as heart rate (HR), respiration rate (RR), oxygenation (SpO2), and blood pressure (BP) through their physiological signals [1-3].

Although these techniques have greatly increased the capabilities of not only physicians but also untrained users, they still require contact with the patient through the use of sensors placed on the patient. However, researchers have introduced a technology that has shown promising results in this area: remote photoplethysmography (rPPG) [4-7]. This technology uses images from a regular camera combined with machine learning algorithms to estimate the same vital signs as contact-based methods.

The information obtained with rPPG indicates changes in skin tissue blood volume that are influenced by heart activity. Frame-to-frame variations in an RGB camera make it possible to see how changes in blood volume and blood vessel wall movement affect light reflection [4]. One key advantage of rPPG is that it is non-invasive and can be performed remotely, making it well-suited for monitoring patients in a variety of settings, including hospitals, homes, and the field. Additionally, rPPG is relatively low-cost, making it accessible to a wide range of healthcare providers [1].

However, there are a number of difficulties in obtaining the best rPPG signal. Low illumination, excessive head movement, and low-end camera device characteristics such as frame rate and resolution can all lead to signal distortions. Moreover, pigmentation of the skin has been a major concern due to the light-based nature of the rPPG technique and the lack of studies involving participants with different skin tones.

Frame-to-frame extraction, region of interest (ROI) recognition, signal processing, and vital sign estimate are the four steps needed to develop an rPPG system. These steps are divided into blocks, all of which are based on well-established, state-of-the-art concepts such as face detection, landmark positioning, frequency spectrum analysis, and digital signal processing.

First, the video obtained from the camera is separated into several frames, with the number of frames per second (FPS) denoted as the frame rate. The minimal frame per second (FPS) necessary to detect rapid changes in the heart cycle is one restriction of rPPG devices. As the heart rate increases, so does the required FPS, but this is usually not an issue for most existing smartphone cameras.

The next step is to find ROIs, which are usually extracted from the user's face and theoretically compared to small sensors placed on the face. While different authors may place their ROIs in different places, a typical strategy is to use face-tracking algorithms such as the Viola-Jones method to find face areas in each video frame [5]. The RGB color space's pixel intensity components are extracted after the ROIs have been chosen. Additionally, to produce a red, blue, and green component for each frame that makes up the raw signals, the RGB components are spatially averaged over all of the pixels in the ROI.

Assessing heart rate, signal processing, and plane-orthogonal-to-skin (POS)

Signal processing is conducted on the raw signal, which is also referred to as the "rPPG core." The rPPG core has been the topic of numerous studies over the last decade, yielding numerous approaches for extracting a clean rPPG signal from the RGB components. There are many approaches in the literature to achieve this, such as those that rely on blind source separation (BSS) methods [6,7]. These techniques, which use different criteria to separate temporal RGB traces into uncorrelated or independent signal sources, can retrieve information by de-mixing raw signals into different sources, including principal component analysis (PCA)-based and independent component analysis (ICA)-based techniques. Other researchers have attempted to increase signal quality by converting the color space to a chrominance-based domain [8].

Wang et al. [9] provided a novel option to transform RGB components into rPPG signals, the "plane-orthogonal-to-skin" (POS) algorithm, in more recent work. In a nutshell, the POS approach attempts to remove intensity variations by projecting RGB components onto a plane orthogonal to a normalized skin tone vector. The projections are referenced in a 2D signal, which is then combined into a 1D signal, which is one of the input signal dimensions weighted by an alpha parameter. The alpha parameter is the quotient of each signal's standard deviations. Despite the successful results achieved by all of the aforementioned authors, none of them have evaluated or investigated the impact of skin tone on the results.

In this paper, we propose to evaluate rPPG Software Development Kit (SDK) version 3.0 against medical devices for participants with different skin tones. This SDK, which can be integrated into Android and iOS mobile apps as well as web applications, provides physiological assessment estimations for a number of vital signs, with heart rate and heart rate variability (HRV) chosen as the major characteristics for this benchmark. The initial hypothesis of our methodology is to achieve a heart rate error within 3 bpm, HRV-inter-beat interval (IBI) within 50 ms, and HRV-standard deviation of normal to normal heartbeats (SDNN) within 15 ms. Additionally, this paper seeks to address the assumption that rPPG technology suffers from bias when used on people with dark skin tones.

This article was previously posted to the medRxiv preprint server on April 3, 2023.

## Materials and methods

**Figure 1:**
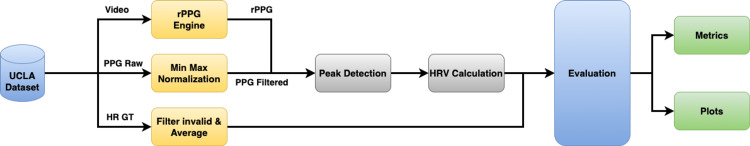
Block diagram of the study methodology UCLA: University of California, Los Angeles, rPPG: remote photoplethysmography, PPG: photoplethysmography, HRV: heart rate variability, HR: heart rate, GT: ground truth

In this section, we present an overview of rPPG technology, covering its most important aspects and blocks. Additionally, the methodology used in this study will be addressed. Figure 1 shows the block diagram of the proposed study. All videos in the dataset are synchronized with ground truth (GT) heart rate and PPG signals extracted from a commercial pulse oximeter placed on the subject's finger.

rPPG processing

**Figure 2:**
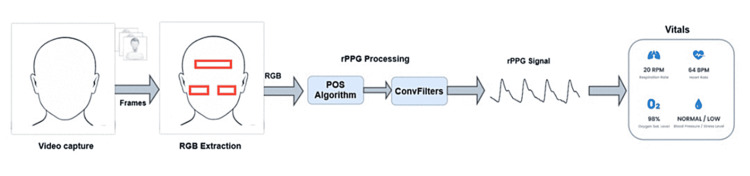
rPPG extraction method rPPG: remote photoplethysmography, RGB: red, green, and blue, POS: proof of stake, ConvFilters: convolutional filters

In this section, a detailed description of the rPPG core method will be provided. The goal of this method is to obtain an ideal rPPG signal that is as clean as feasible and has the same physiological information as a PPG signal from a contact sensor. This signal reflects the cardiac cycle and body hemodynamics. The method is depicted in Figure 2.

The RGB components were extracted from the ROIs using the landmark detection algorithm from the OpenCV library [10]. In a recent paper [11], we recommended using three ROIs from the forehead, left cheek, and right cheek, which performed best in internal experiments. Once the raw signal was collected, a version of the POS algorithm proposed by Wang et al. [9] is applied, and the resulting signal is further sent to a filtering stage based on convolutional filters (ConvFilters). This stage aims to enhance the quality of the rPPG signal by denoising it as a sinusoidal wave [12].

POS algorithm

The POS algorithm, originally developed by Wang et al. [9], aims to combine RGB channels into a single-channel rPPG signal. According to the authors, the input RGB signal channels are combined on the time interval (t) as shown in Equations 1 and 2:

$U(t) = G_{n}(t) - B_{n}(t)$ (1)

$V(t) = G_{n}(t) - R_{n}(t) + B_{n}(t) -R_{n}(t)$ (2)

The subscript n stands for normalized, and it represents the instant color values divided by the color channel's mean value. The rPPG signal for this interval is built as shown in Equation 3:

$rPPG(t,t_{i}) = U(t) + \alpha(t_{i})V(t)$ (3)

α is the ratio of the standard deviations of U(t) and V(t) across the interval.

Convolutional filter

The purpose of this stage is to improve signal quality by reducing noise as much as possible, which is accomplished by using a convolutional filter (ConvFilter). Convolution is performed between the input single-channel signal, s_orig, which is recovered following the POS method, and a template that represents a single heartbeat peak of the same signal in the ConvFilter. To build the template, average portions of the s_orig signal around the discovered peaks. Furthermore, because the s_orig signal could contain some noise, a band-pass filter with a bandwidth ranging from 0.7 Hz to 7.0 Hz was used to aid in peak detection. Convolution Equation 4 or the equivalent correlation Equation 5 with this template t[k] yields the cleaner "s_heart" signal:

$$\label{eqn:s_heart_1} s\_heart[n] = \frac{1}{2K+1} \sum\limits_{k=-K}^{K} s\_orig[n-k]t[-k]$$

(4)

$$\label{eqn:s_heart_2} s\_heart[n] = \frac{1}{2K+1} \sum\limits_{k=-K}^{K} s\_orig[n+k]t[k]$$

(5)

UCLA dataset

The dataset comprises 98 subjects and 489 videos of various skin tones, ages, genders, ethnicities, and races. The skin tone of each subject was determined using the Fitzpatrick (FP) skin type scale [13,14], which ranges from 1 to 6. For each subject, five videos of approximately one minute were recorded at 30 frames per second (about 1,800 frames), resulting in uncompressed videos with a total size of 2 gigabytes. All videos in the dataset are synchronized with ground truth heart rate and PPG signals extracted from a pulse oximeter placed on the subject's finger.

Metrics

The purpose of this work, as previously stated, is to test the suggested approach of remote health screening utilizing rPPG signals retrieved from video data. To assess the performance, heart rate and HRV features were used. Heart rate is a well-established parameter that is familiar to most people and ranges from low to high. In contrast, HRV may sound unfamiliar to many, but it can provide important insights into a person's health. HRV-SDNN was used to assess health status, while HRV-low frequency (LF) and HRV-high frequency (HF) were correlated to the autonomic nervous system through the sympathetic and parasympathetic branches [13,15,16].

As heart rate was analyzed, the GT value provided in the dataset was compared to the estimated heart rate. For the HRV assessment, the comparison was made with the HRV features calculated using the PPG GT signal. Thus, physiological characteristics were extracted and analyzed from both touch and distant signals. The basic instrument for calculating such properties was the RR interval, commonly known as the pulse-to-pulse interval. It is the time difference in milliseconds (ms) between two peaks (Equation 6). In addition, the following features were employed. The time domain variables are inter-beat interval (Equation 7), root mean square of successive differences between normal heartbeats (RMSSD) (Equation 8), and standard deviation of normal to normal heartbeats (SDNN) (Equation 9). In the frequency domain, the power from the low-frequency band (LF) (0.04; 0.15) Hz and the high-frequency band (HF) (0.15; 0.4) Hz was measured. All comparisons were conducted in terms of mean absolute error (MAE), mean error (ME), and 2D plots.

$$\label{eqn:RR_interval} RR(i) = \frac{peak(i) - peak(i+1)}{sampling frequency} \times 1000.0$$

(6)

$$\label{eqn:IBI} IBI = mean(RR)$$

(7)

$$\label{eqn:RMSSD} RMSSD = \sqrt{\frac{1}{N-1} \times \sum\limits_{j=1}^{N} (RR(i) - RR(i+1))^2}$$

(8)

$$\label{eqn:SDNN} SDNN = \sqrt{\frac{1}{N-1} \times \sum\limits_{j=1}^{N} (RR(i) - mean(RR))^2}$$

(9)

Video recordings from the UCLA dataset [13] were used in this work to extract physiological data from people. Using the suggested technique outlined previously, each video is processed to produce the rPPG signal. Furthermore, the information obtained through the rPPG signal is compared with the ground truth heart rate extracted from a commercial oximeter placed on the subject's finger, as well as HRV features extracted from the contact PPG signal. The goal of this study is to not only benchmark the overall results but also assess the impact within certain groups based on skin tone and gender.

## Results

Firstly, after extracting the rPPG signals, it was necessary to remove some samples. However, the criteria differed for HR and HRV due to their different sources. Since HR was obtained directly from the sensor and HRV was calculated from the PPG signal, some samples might be useful for one benchmark, but not for the other. The following issues were identified: some samples had no GT HR and were characterized by constant values of 255 and 129, which caused their exclusion from the dataset.

In addition, some samples were manually checked, and three cases were observed: poor PPG GT quality, PPG GT discontinuity (probably caused by interference or sensor displacement), and increasing heart rhythm. Samples from the first two cases were removed from the HRV benchmark, but not from HR, as long as the GT HR presented reasonable values (HR < 200 bpm).

Lastly, some samples presented an irregular heart rhythm, with an increase of up to 10 bpm at some point during the experiment. This might be caused by several factors such as not being at rest when taking the measurement, participants speaking or moving during the reading, or deliberately increasing heart rate. Additionally, a less likely cause could be a correlation with cardiac diseases. These samples were not removed from the dataset, as this work understands that the technology should be able to pick up these changes as well. However, this represents a bigger challenge.

**Figure 3:**
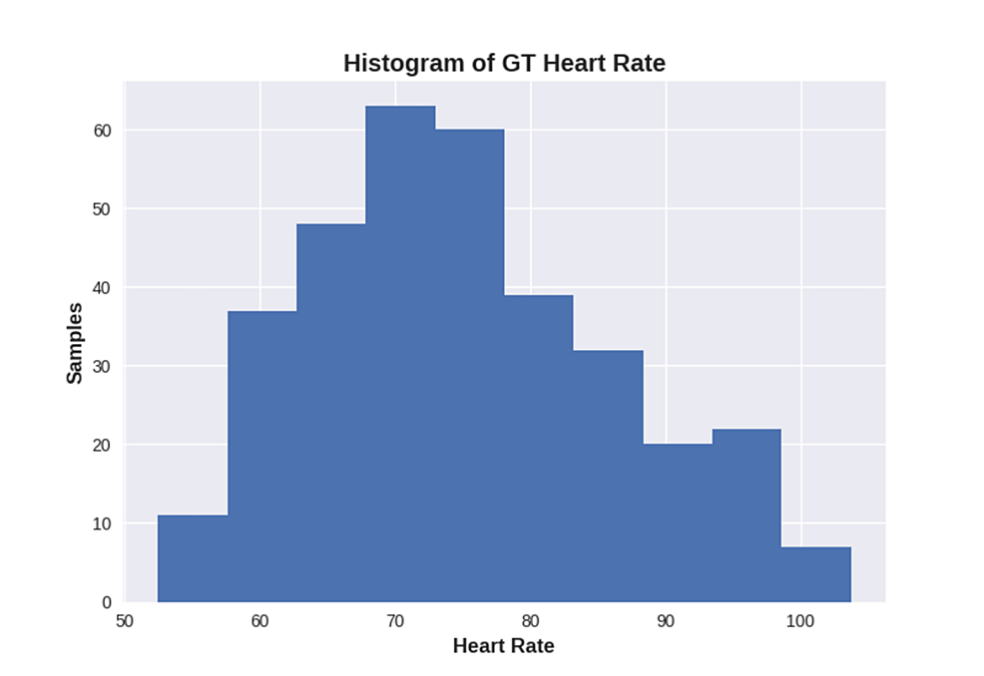
Ground truth heart rate distribution GT: ground truth

Additionally, samples that could not have their HR estimated were also removed, as this work understands that there is no point in outputting a value if there is no certain confidence. As a result, the dataset for HR benchmarking contained 339 samples from 90 unique participants, while the HRV dataset contained 332 and 94 samples, respectively. Figure 3 shows a histogram of GT HR for all remaining samples [13].

For this study, subgroups were created to enhance the understanding of the results. Firstly, we evaluated the results from a signal quality perspective in terms of signal-to-noise ratio (SNR), measured in decibels (dB). Three groups were used: SNR > 5 dB, which is classified as the minimum required; SNR > 8 dB, which is classified as optimal signals; and SNR > 10 dB, which is considered perfect signal quality. Regarding skin tone groups, based on the Fitzpatrick skin type [14], three other groups were considered: light skin tones related to values 1 and 2 of the scale; medium skin tones, consisting of skin tones with values 3 and 4 of the scale; and dark skin tones, consisting of skin tones 5 and 6 of the scale. Additionally, this study chose to split gender into two groups: male and female. For the skin tone and gender groups, no exclusion was performed based on minimum SNR. Table 1 shows the results for heart rate estimation for all samples as well as each one of the subgroups in terms of mean absolute error and mean error.

**Table 1:** Heart rate evaluation across different subgroups bpm: beats per minute, MAE: mean absolute error, ME: mean error, SNR: signal-to-noise ratio, LF: low-frequency band, HF: high-frequency band

Group	Number of samples	Number of subjects	Metrics (bpm)	
			MAE	ME
All samples	339	90	3	2
SNR > 5	326	88	3.01	2
SNR > 8	308	87	3.02	2.03
SNR > 10	248	80	2.89	1.86
Fitzpatrick 1 and 2	104	27	2.53	1.58
Fitzpatrick 3 and 4	183	48	3.05	1.82
Fitzpatrick 5 and 6	52	15	3.79	3.46
Male	116	29	3.24	2.01
Female	223	61	2.88	1.99

**Figure 4:**
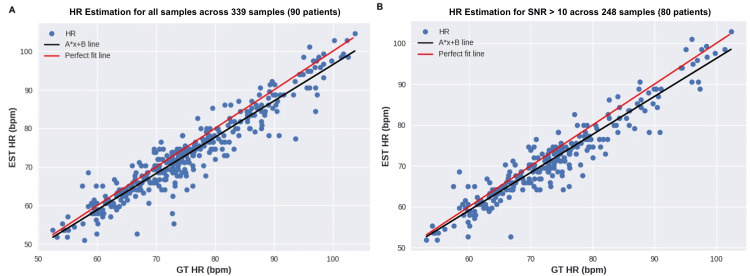
Scatterplot of HR across all samples and optimal signal quality HR: heart rate, SNR: signal-to-noise ratio, bpm: beats per minute, EST: exercise stress test, GT: ground truth

Additionally, Figure 4 presents two scatterplots: Figure 4A with all available samples (after filtering) and Figure 4B, which only includes samples with perfect signal quality (SNR > 10 dB). The figures depict the best-fit line (black) and the perfect line (red).

**Figure 5:**
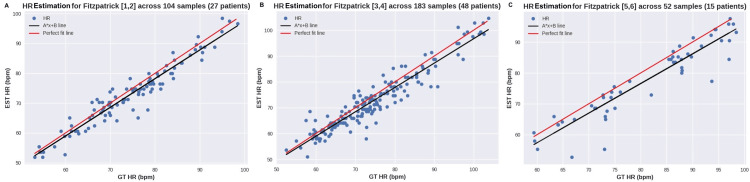
Scatterplot of HR for various skin tones according to the Fitzpatrick scale A: skin tones 1 and 2, B: skin tones 3 and 4, C: skin tones 5 and 6 HR: heart rate, EST: exercise stress test, bpm: beats per minute, GT: ground truth

Additionally, Figure 5 shows a similar plot for different skin tone groups. Figure 5A depicts people with light skin tones, defined by Fitzpatrick scale values 1 and 2, while Figure 5B displays the impact on medium skin tones for Fitzpatrick values 3 and 4. Finally, Figure 5C presents the results for dark skin tones, categorized as 5 and 6 on the Fitzpatrick scale [13].

**Figure 6:**
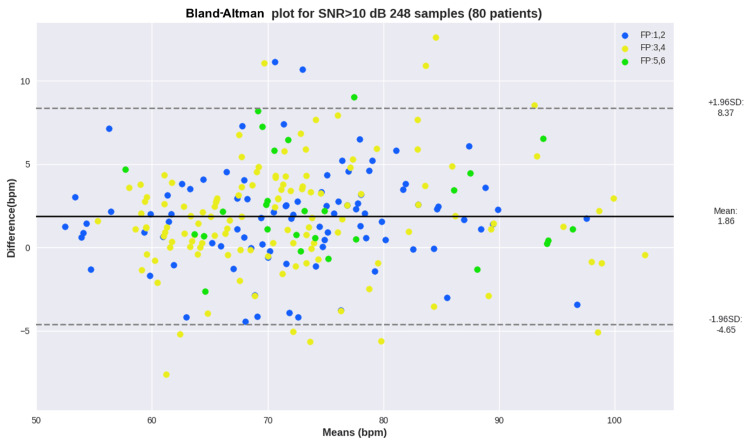
Bland-Altman plot for heart rate estimation across samples with SNR > 10 dB SNR: signal-to-noise ratio, bpm: beats per minute, FP: Fitzpatrick, dB: decibels, SD: standard deviation

Despite the scatterplots being a fair representation of the distribution of errors, Bland-Altman plots bring the results in terms of the mean value versus the difference between the ground truth (GT) and estimation. Figure 6 shows the Bland-Altman plot for heart rate estimation across samples with SNR > 10 dB, where the skin tones were highlighted for each group.

Although heart rate is a reliable feature to benchmark physiological data extraction, heart rate variability (HRV) can provide a more in-depth analysis of someone's current health status. As stated previously, both time and frequency domain HRV features can reveal important health insights. Similar to the heart rate analysis, Table 2 and Table 3 show an overview of the results for the time and frequency domains, respectively, for all samples as well as for each subgroup.

**Table 2:** Heart rate variability time domain evaluation across different subgroups SNR: signal-to-noise ratio, LF: low-frequency band, HF: high-frequency band, MAE: mean absolute error, ME: mean error, IBI: inter-beat interval

Group	Samples	Subjects	Metrics (ms)					
			MAE			ME		
			IBI	Standard deviation of normal to normal heartbeats	Root mean square of successive differences between normal heartbeats	IBI	Standard deviation of normal to normal heartbeats	Root mean square of successive differences between normal heartbeats
All samples	332	94	9.49	14.35	22.49	-5.29	1.6	1.38
SNR > 5	298	89	7.87	13.57	21.96	-5.06	1.87	2.4
SNR > 8	265	84	7.14	12.88	20.79	-4.92	2.14	3.14
SNR > 10	207	75	6.94	12.59	21.19	-5.03	1.82	3.69
Fitzpatrick 1 and 2	96	27	8.91	14.53	24.14	-7.72	2.96	4.39
Fitzpatrick 3 and 4	173	48	8.6	14.13	22.37	-4.25	1.16	1.15
Fitzpatrick 5 and 6	63	19	12.82	14.69	20.31	-4.42	0.71	-2.58
Male	111	30	9.62	15.54	21.29	-4.67	3.88	1.23
Female	218	63	9.43	13.89	23.34	-5.55	0.44	1.43

**Table 3:** Heart rate variability frequency domain evaluation across different subgroups SNR: signal-to-noise ratio, LF: low-frequency band, HF: high-frequency band, MAE: mean absolute error, ME: mean error

Group	Samples	Subjects	Metrics (s2/Hz)			
			MAE		ME	
			LF	HF	LF	HF
All samples	329	94	16.2	34.09	-5.26	9.67
SNR > 5	295	89	15.97	33.8	-5.57	8.78
SNR > 8	262	84	15.16	32.71	-5.78	9.28
SNR > 10	205	75	15.66	33.49	-6.48	9.32
Fitzpatrick 1 and 2	96	27	17.34	28.02	-4.68	8.53
Fitzpatrick 3 and 4	170	48	14.62	35.93	-6.2	7.39
Fitzpatrick 5 and 6	63	19	18.69	38.38	-3.6	17.56
Male	108	30	15.84	31.76	-1.9	1.39
Female	218	63	16.37	35.43	-7.19	13.69

**Figure 7:**
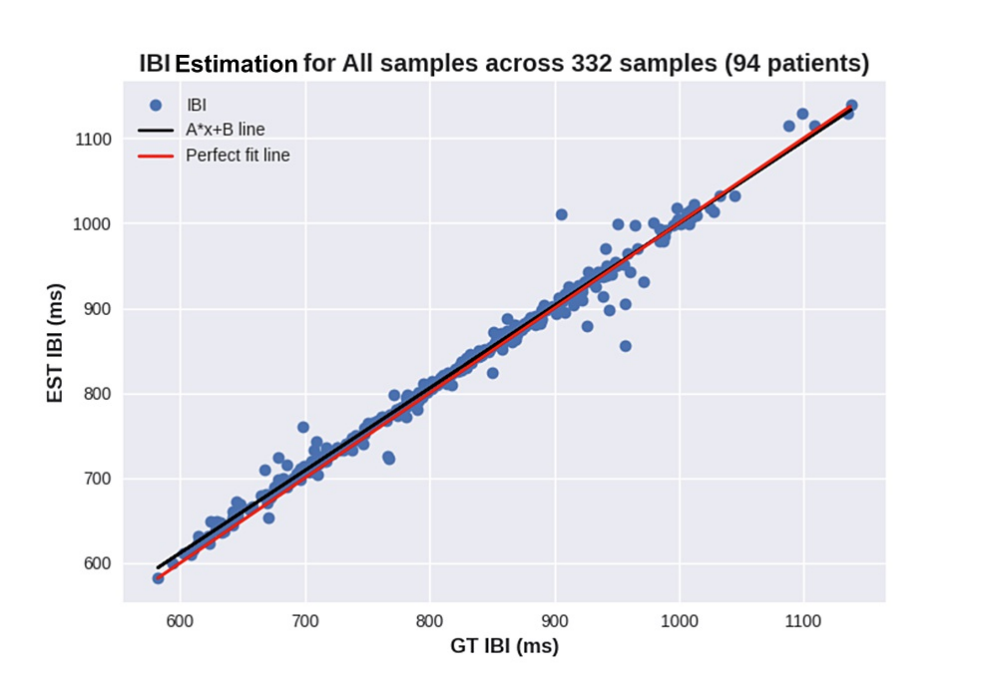
Scatterplot of inter-beat intervals across all samples IBI: inter-beat interval, GT: ground truth, EST: exercise stress test

This work proposes two main analyses of heart rate variability (HRV) for a more in-depth assessment of participants' health status. Firstly, an analysis of the inter-beat interval (IBI) across all samples is presented in Figure 7, which highlights the mean IBI and thus the cardiac rhythm.

**Figure 8:**
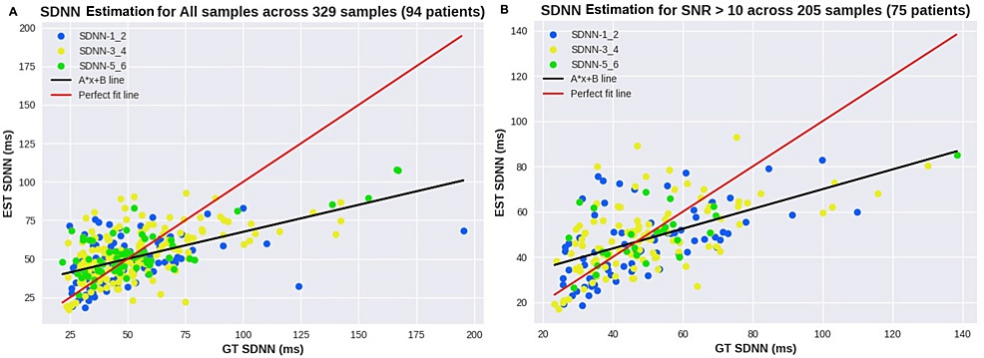
Scatterplot of SDNN across all samples and optimal signal quality SDNN: standard deviation of normal to normal heartbeats, SNR: signal-to-noise ratio, EST: exercise stress test, GT: ground truth

**Figure 9:**
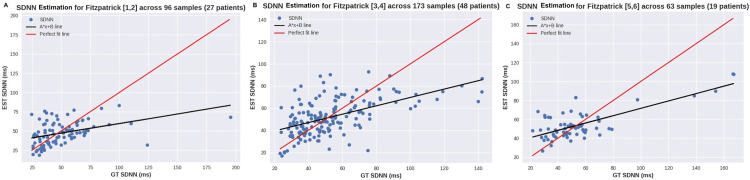
Scatterplot of SDNN for various skin tones according to the Fitzpatrick scale A: skin tones 1 and 2, B: skin tones 3 and 4, C: skin tones 5 and 6 SDNN: standard deviation of normal to normal heartbeats, EST: exercise stress test, GT: ground truth

Secondly, the study has chosen SDNN as the main HRV metric for in-depth analysis, as SDNN has been used as a fitness and health score. Figure 8 shows a scatterplot of SDNN across all samples (Figure 8A) as well as for samples with perfect signal quality (Figure 8B). For these plots, the skin tone groups were highlighted in different colors to identify the impact of skin tone on the presented errors. Similarly, Figure 9 shows the scatterplots for each skin tone group separately [13].

**Figure 10:**
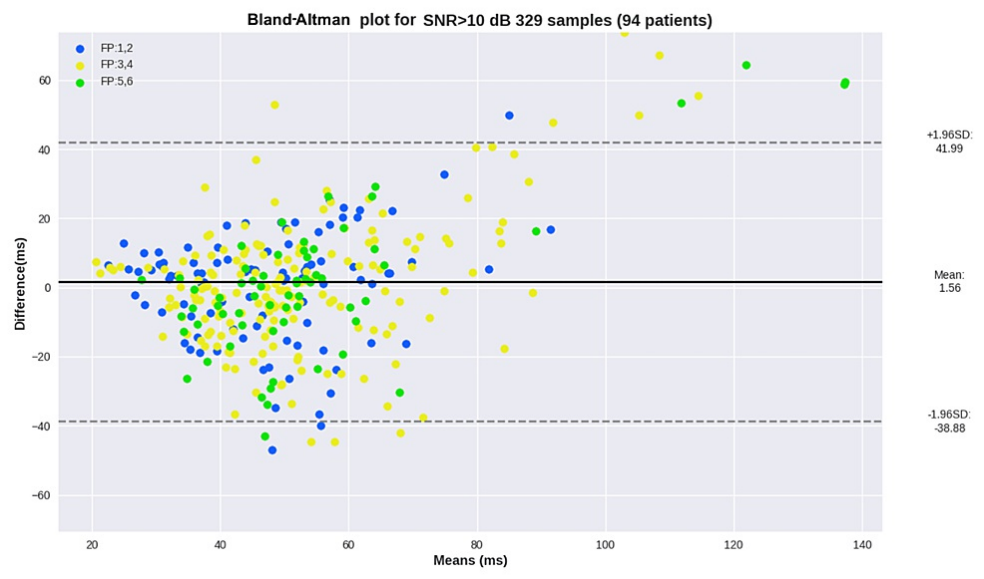
Bland-Altman plot for SDNN estimation across samples with SNR > 10 dB SDNN: standard deviation of normal to normal heartbeats, SNR: signal-to-noise ratio, FP: Fitzpatrick, SD: standard deviation

Lastly, the Bland-Altman plot for SDNN across samples with SNR > 10 dB is presented in Figure 10. As with the previous plots, skin tone groups based on the Fitzpatrick scale are highlighted in different colors to facilitate the identification of samples with higher errors.

In this work, video files of 98 participants were extracted from the University of California, Los Angeles (UCLA) dataset, resulting in a total of 498 videos, each lasting one minute and captured at a frequency of 30 Hz. For each video, contact PPG and heart rate were synchronously measured using a pulse oximeter, and the skin tone was classified using the Fitzpatrick scale. After verifying the integrity of the data and removing erroneous samples, around 330 samples from 90 patients were retained.

## Discussion

In the past decade, rPPG technology has grown significantly with the advancement of computational power and the use of machine learning algorithms in various fields, including the biomedical field, where researchers have been improving non-invasive and contactless techniques for measuring physiological data. However, the increasing demand for benchmark datasets to evaluate these methods has also highlighted some concerns [12]. Despite these challenges, Wang et al. [13] recently proposed the largest known rPPG dataset, which includes a variety of participants with different skin tones. The benchmark was conducted in terms of heart rate and heart rate variability, which are well-established metrics used to assess an individual's health status. This work proposed dividing the samples into subgroups to enhance the analysis perspective. First, in terms of signal quality, three groups were created for minimum, optimal, and perfect conditions. Additionally, the samples were divided into three skin tone groups based on the Fitzpatrick scale, as well as two gender groups.

The first experiment aimed to evaluate the accuracy of the algorithm for heart rate estimation. Table 1 shows that across all samples, the MAE error was 3 bpm, which meets the initial hypothesis. Moreover, the performance was stable across samples with SNR > 5 and 8 dB, as can be seen in Figure 4. This indicates that even with lower signal quality, the algorithm can still estimate HR accurately. When taking into account signals with perfect quality, there is a slight improvement to 2.89 bpm. It is worth noting that more than half of the samples had perfect signals, as well as 80 out of 90 patients. Although the dataset was collected in a good setting, signal quality is also a direct result of power on the calibration of light changes and movement compensation to enhance the extracted signal. Concerning skin tone groups, most of the participants (48) were in the medium skin tone group (FP 3 and 4), which achieved an MAE of 3.05 bpm. The dark skin tone group had a slightly higher MAE of 3.79 bpm, but it was less than 1 bpm from the original mark. Through the comparison between Figure 5C and Figure 5B, it is possible to see the difference in the relationship between the 1:1 line and the best-fit line, where in the former, the greatest differences remain when the estimated value was smaller than the actual value.

Despite the initial results showing a slight decrease in performance, this may be due to other factors, such as signal quality and an increase in heart rate throughout the reading. The Bland-Altman plot presented in Figure 6 corroborates this hypothesis, as it shows all readings with SNR 10+, and no correlation can be seen between the skin tone group and errors. The blue dots represent light skin tones, yellow for medium, and green for dark skin tones. Additionally, the plot shows a mean error of 1.86 bpm across all the readings with SNR > 10 dB. Moreover, out of the 98 participants, 61 were females and 29 were males. There was no major discrepancy in the results between the two gender groups, indicating no bias toward gender.

Regarding the HRV experiments, this work extracted results from five different features, three of which are from the time domain (IBI, SDNN, and RMSSD), and two are from the frequency domain (LF and HF). In the overview results presented in Table 2, it is possible to see that IBI displays the lowest error range within 10 ms, which is a significant result and far exceeds the initial hypothesis of 50 ms. If we consider that IBI ranges from 500 to 1,500, 10 ms would reflect an average error of 1 bpm. Moreover, SDNN, as previously stated, has been used to determine the fitness score and shows an average error of 14 ms, which also meets the proposed value of 15 ms. Normal ranges of SDNN vary from 30 to 150.

Through the analysis of HRV, conclusions can be drawn about the previous experiment as well, since HRV can provide additional insight into the cardiac rhythm. While IBI displays an almost perfect correlation, as observed in Figure 7, SDNN shows some discrepancies, often related to high SDNN values as can be seen in Figure 8A, Figure 9A, and Figure 9C, where the ground truth (GT) SDNN was above 100 ms. These values are highly associated with the increasing heart rhythm previously discussed, which can be hidden in some metrics but clear in the standard deviation. While these values are not wrong in terms of metrics, they do not reflect the rhythm conditions, most likely because they were not at rest. Thus, these samples should be considered outliers, and their exclusion would result in a decrease in MAE SDNN to around 9 ms.

Regarding subgroups, the overview results show that MAE was consistent across all Fitzpatrick groups (around 14 ms). The plots show sparse estimations, and once again, no correlation between the skin tone group and the errors can be seen. The Bland-Altman analysis in Figure 10 shows a mean SDNN error across samples with SNR 10+ of 1.56 ms, and most differences remain within +-20 ms.

Additionally, an increase in the MAE of the male group to 15.54 ms can be observed. This phenomenon can be explained because most of the samples with an increasing heart rhythm were from males, which directly affects the SDNN and RMSSD metrics. Regarding RMSSD, although most of the MAE results remain stable around 22 ms, it is noticeable that the lowest MAE is observed in the dark skin tone group, with 20.31 ms.

Finally, acceptable results were also obtained in the frequency analysis. The normalized power of the low- and high-frequency bands was extracted from contact and remote signals for comparison. As shown in Table 3, the MAE for the low-frequency band is within 16 s2/Hz, while for the high-frequency band, it is within 38 s2/Hz. The normal ranges for LF and HF are 0-70 s2/Hz and 10-175 s2/Hz, respectively. Through the LF and HF features, correlations with the autonomic nervous system can be inferred.

Limitations

While many efforts have been made to collect rPPG datasets for more accurate physiological sensing, these datasets may have limitations, such as a small number of subject participants and biases toward certain demographic groups. Furthermore, few studies have explored the technology's boundaries and limitations, such as its accuracy in darker skin tone populations, which remains largely unexplored due to the lack of proper datasets. For instance, Dasari et al. [12] proposed a dataset that only contains dark skin tones, but the actual videos are not shared, only the color space values of the skin region of interest.

## Conclusions

In this paper, a benchmark of our methodology using the largest known rPPG dataset was proposed. Approximately 340 video files from 90 patients were analyzed, and from these, rPPG signals, heart rate, and heart rate variability were extracted and compared to ground truth information obtained from a pulse oximeter. This study aimed to address the lack of results of rPPG technology in a population of individuals with different skin tones, as well as to mitigate concerns about the accuracy of the method in people with dark skin tones. Heart rate and heart rate variability were chosen as the main features to evaluate similarity due to their ease of extraction, well-known features, and ability to potentially reflect health and fitness insights about the user. The analysis was carried out within subgroups based on skin tone, with three groups created based on the Fitzpatrick scale. The results have shown that the heart rate meets the initial hypothesis of a mean absolute error of 3 bpm. Within the skin tone subgroups, no significant performance glitches were observed. Moreover, the HRV results have shown an almost perfect correlation with IBI, with a mean absolute error within 10 ms (around 1 bpm). Similarly, SDNN has shown acceptable results within 14 ms, although some samples presented an increasing cardiac rhythm during the reading, which drove the HRV metrics toward deceptive values.

The study has shown that our methodology meets acceptable agreement levels for mean absolute error for HR, HRV-IBI, HRV-SDNN, HRV-RMSSD, HRV-LF, and HRV-HF. Furthermore, the experiments have shown that skin tone had no impact on the results, which all remained within the same range. Moreover, this work has demonstrated through its results that rPPG technology can be used and has no significant accuracy impact on dark-skinned people. This demonstrates that consumers who want to understand their general health and wellness can adopt the suggested methods.
